# The Adam–Gibbs relation and the TIP4P/2005 model of water

**DOI:** 10.1080/00268976.2018.1471230

**Published:** 2018-05-10

**Authors:** Philip H. Handle, Francesco Sciortino

**Affiliations:** Department of Physics, Sapienza University of Rome, Roma, Italy

**Keywords:** Adam–Gibbs, water, glass, PEL

## Abstract

We report a numerical test of the Adam–Gibbs relation for the TIP4P/2005 model of water. The configurational entropy is here evaluated as the logarithm of the number of different basins in the potential energy landscape sampled in equilibrium conditions. Despite the non-monotonic behaviour which characterise the density dependence of the diffusion coefficient, the Adam–Gibbs relation is satisfied within the numerical precision in a wide range of densities and temperatures. We also show that expressions based on the excess entropy (the logarithm of the number of sampled microstates in phase space) fail in the region of densities where a tetrahedral hydrogen bond network develops.

## GRAPHICAL ABSTRACT

**Figure:**
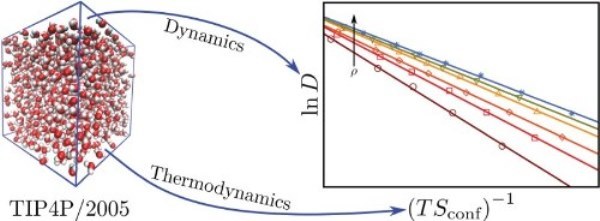

## 1. Introduction

One of the most intriguing phenomena in condensed matter physics is the glass transition. The temperature (*T*) at which it occurs is conventionally defined at the *T* at which viscosity (*η*) reaches values of the order of $10^{12}$ Pa s. Such high viscosities render flow impossible on reasonable observation timescales and hence the liquid turns into a disordered solid. The tendency of liquids to form disordered solids, i.e., glasses, on cooling varies. Protein solutions typically form disordered arrested states readily [1], making crystallisation the hard problem [2]. Others such as silica are both fairly easily obtained as a crystalline solid or as a glass [3]. Finally, there are substances that tend towards crystallisation, challenging the creativity of experimentalists if a glass is desired. An example of such a bad glass former is water. Several methods had to be devised to obtain it in its glassy form [4–7]. Besides the tendency of a material to form a glass, an interesting feature is the change of *η* or of the diffusion coefficient *D* upon cooling before reaching the glassy state. The way *η* or *D* change with *T* conveys information not only on the possibility to shape the supercooled liquid on its way to the glass, but also on the physical process controlling the slowing down of the dynamics. Typically two types of liquids are discerned. *Strong* liquids show an Arrhenius-type behaviour, i.e., lnη vs. 1/*T* is linear, whereas *fragile* liquids display Super-Arrhenius behaviour, described by the Vogel–Fulcher–Tammann (VFT) relation [8]:

(1) $$\eta =\eta_{\infty }e^{BT_{0}/(T-T_{0})}.$$

Here *B*, $T_{0}$ and $\eta_{\infty }$ are (path dependent) constants.

An intriguing connection between thermodynamics and the VFT relation for dynamical quantities is offered by the Adam–Gibbs (AG) relation [9]:

(2) $$\eta =\eta_{\infty }e^{A/(TS_{\mathrm{conf}})}.$$

Here $S_{\mathrm{conf}}$ indicates the configurational entropy, proportional to the logarithm of the number of distinct relevant liquid configurations. The idea behind Equation (2) is based on the concept of ‘cooperatively rearranging regions’ (CRR), spatial regions in which relaxation processes take place cooperatively. The activation energy for such relaxations is assumed to be extensive in the number of atoms or molecules that make up the CRR. The increase in the size of the CRR becomes thus associated to a decrease of the configurational entropy and responsible for the slowing down of the dynamics on supercooling. Numerical studies have attempted to identify the CRR from atomistic configurations. For the case of water see for example Ref. [10]. If $S_{\mathrm{conf}}$ vanishes at finite *T*, the AG model is consistent with approaches predicting an underlying (thermodynamic) phase transition as origin of the divergence of relaxation time. If $TS_{\mathrm{conf}}$ can be linearly expanded around the temperature $T_{0}$ at which it vanishes as $TS_{\mathrm{conf}}=\left(A/B\right)\cdot \left(T-T_{0}\right)/T_{0}$, (where now $A/BT_{0}$ is the coefficient of the linear expansion) Equation (2) coincides with Equation  (1). In this case $T_{0}$ is called the Kauzmann temperature, being the *T* where $S_{\mathrm{conf}}=0$. Equations (1) and (2) can also be written in terms of the diffusion constant *D* or other structural relaxation times *τ*. Due to the possible decoupling between *D* and *η* the parameters in the equations are in some cases sensitive to the chosen observable. For a critical exam of the AG model see for example [11].

The potential energy landscape (PEL) approach identifies $S_{\mathrm{conf}}$ in the liquid state as the logarithm of the number of distinct basins sampled on the potential energy surface [12], each of them conventionally associated to the minimum potential energy of the basin, the so-called inherent structure (IS). The PEL approach offers also a consistent way to numerically evaluate $S_{\mathrm{conf}}$ [13–16], which avoids the approximation of $S_{\mathrm{conf}}$ as the difference between the liquid and the solid entropy commonly adopted in experiments [17–19]. PEL-based studies have offered the possibility to check the validity of the AG relation in several model potentials [20–27]. In most cases, it has been shown that a representation of ln⁡D or ln⁡η vs. $(TS_{\mathrm{conf}}{)}^{-1}$ is consistent with the numerical results.

Very recently we have reported a numerical study of the statistical properties of the PEL for the TIP4P/2005 model of water [28]. We have shown that a Gaussian PEL properly describes the simulation data, reproducing the thermodynamic anomalies characteristic of water and predicting the existence of a liquid-liquid critical point. In this contribution we expand the landscape analysis to dynamics, testing the validity of the Adam–Gibbs relation for TIP4P/2005, the most accurate classical water model to date [29,30]. For completeness, we also compare the *T* and density (*ρ*) dependence of the diffusion coefficient with other propositions relating the excess $S_{\mathrm{exc}}$ and the two-point entropy with dynamics [31].

## 2. Simulation details

### 2.1. NVT simulations

We perform NVT simulations of 1000 TIP4P/2005 molecules in a cubic box utilising GROMACS 5.1.2 [32] with a leap-frog integrator using a timestep of 1 fs. The temperature is controlled using a Nosé-Hoover thermostat [33,34] with a time constant of 0.2 ps. For the coulombic interactions we use a particle mesh Ewald treatment [35] with a Fourier spacing of 0.1 nm. For both the Lennard-Jones and the real space Coulomb interactions, a cut-off of 0.85 nm is used. Lennard-Jones interactions beyond 0.85 nm have been included assuming a uniform fluid density. Finally, we maintain the bond constraints using the LINCS (Linear Constraint Solver) algorithm [36] of 6th order with one iteration to correct for rotational lengthening. We investigate 14 different densities from 0.9 to 1.42 g/cm^3^ and seven different *T*s between 200 and 270 K. Very long equilibration runs (up to 100 ns) followed by equally long production runs have been performed.

### 2.2. Diffusivity

To evaluate the diffusion coefficient *D* we use the standard approach employed in molecular simulations [37]. We calculated the mean-square displacement

(3) $$\mathrm{MSD}(t^{\prime })=\left\langle {\left(\vec{r}(t+t^{\prime })-\vec{r}(t)\right)}^{2}\right\rangle$$

for all MD runs. Here the average is over all particles in the system and over different initial times *t*, while $\vec{r}$ indicates the oxygen position of the generic molecule. Since the oxygen is significantly heavier than the hydrogen atoms, the oxygen position provides a good characterisation of the centre of mass. From the long-time limit of the MSD we evaluate *D* via the Einstein relation

(4) $$\lim_{t^{\prime }\to \infty }\mathrm{MSD}(t^{\prime })=6Dt^{\prime }.$$

### 2.3. Entropy

In this study we evaluate five different entropies. The entropy of the liquid $S_{\mathrm{liq}}$, the vibrational entropy $S_{\mathrm{vib}}$, the configurational entropy $S_{\mathrm{conf}}$, the excess entropy of the liquid with respect to the ideal gas $S_{\mathrm{exc}}$ and a two body approximation of the latter $S^{\left(2\right)}$.

$S_{\mathrm{liq}}$ at each state point is calculated from the total energy $E_{\mathrm{liq}}$ and the free energy of the liquid $F_{\mathrm{liq}}$ via

(5) $$TS_{\mathrm{liq}}=E_{\mathrm{liq}}-F_{\mathrm{liq}}.$$

In Ref. [28] we evaluated $F_{\mathrm{liq}}$ of TIP4P/2005 using a series of thermodynamic and Hamiltonian integration steps [37–39] as well as $E_{\mathrm{liq}}$. The vibrational part of the entropy ($S_{\mathrm{vib}}$) is split into two components. A harmonic component $S_{\mathrm{harm}}$ and an anharmonic component $S_{\mathrm{anh}}$. For the evaluation of $S_{\mathrm{harm}}$ at least 30 equally spaced configurations were extracted from the MD production runs. These configurations were minimised using a conjugate-gradient algorithm. The configurations obtained in this way are the inherent structures (IS). For the IS we calculated the density of states by diagonalising the 6N×6N Hessian matrix, thereby obtaining the eigenfrequencies $\omega_{i}$. From these frequencies we calculate $S_{\mathrm{harm}}$ as

(6) $$S_{\mathrm{harm}}=k_{B}\sum_{i=1}^{6N-3}(1-\ln\beta \hbar \omega_{i}).$$

Here ℏ denotes Planck's constant in its reduced form. To evaluate the entropic contribution arising form anharmonicities [28]$S_{\mathrm{anh}}$ we fit the difference between the potential energy and the IS energy along isochores as a polynomial in *T* (with coefficients $c_{i}$), corresponding to

(7) $$S_{\mathrm{anh}}=\sum_{i=2}^{3}\frac{i}{i-1}c_{i}T^{i-1}.$$

Then $S_{\mathrm{vib}}$ and $S_{\mathrm{conf}}$ are calculated *via*

(8) $$S_{\mathrm{vib}}=S_{\mathrm{harm}}+S_{\mathrm{anh}},$$

and

(9) $$S_{\mathrm{conf}}=S_{\mathrm{liq}}-S_{\mathrm{vib}}.$$

The excess entropy with respect to the ideal gas is computed from

(10) $$S_{\mathrm{exc}}=S_{\mathrm{liq}}-S_{\mathrm{id}}.$$

$S_{\mathrm{id}}$ is calculated from the canonical partition function $Z_{\mathrm{id}}$ of a system of non-interacting water-shaped molecules:

(11) $$Z_{\mathrm{id}}=\frac{Z_{T}Z_{R}}{N!}.$$

This partition function can be split in a translational part

(12) $$Z_{T}={\left(V{\left(\frac{2\pi mk_{B}T}{h^{2}}\right)}^{3/2}\right)}^{N}$$

and a rotational part

(13) $$Z_{R}={\left(\frac{1}{2}{\left(\frac{8\pi^{2}k_{B}T}{h^{2}}\right)}^{3/2}{\left(\pi I_{x}I_{y}I_{z}\right)}^{1/2}\right)}^{N},$$

where *m* is the mass of the water molecule, $I_{x}$, $I_{y}$ and $I_{z}$ are the moments of inertia along the three principal axes, $k_{B}$ is Boltzmann's constant and *h* is Planck's constant. The factor 1/2 in front of $Z_{R}$ accounts for the water molecule's $C_{2v}$ symmetry [40]. From this partition function we calculate the free energy of the ideal gas

(14) $$F_{\mathrm{id}}=-k_{B}T\ln Z_{\mathrm{id}}$$

and the ideal gas entropy

(15) $$TS_{\mathrm{id}}=3Nk_{B}T-F_{\mathrm{id}}.$$

Finally we compute a two-body approximation of the translational component of the excess entropy $S^{\left(2\right)}$ from the O-O-pair correlation function g(r) [31]:

(16) $$\frac{S^{\left(2\right)}}{Nk_{B}}=-2\pi \rho \int \{g(r)\ln[g(r)]-g(r)+1\}r^{2}dr,$$

where *ρ* denotes the molecule number density.

## 3. Results

**Figure 1.**
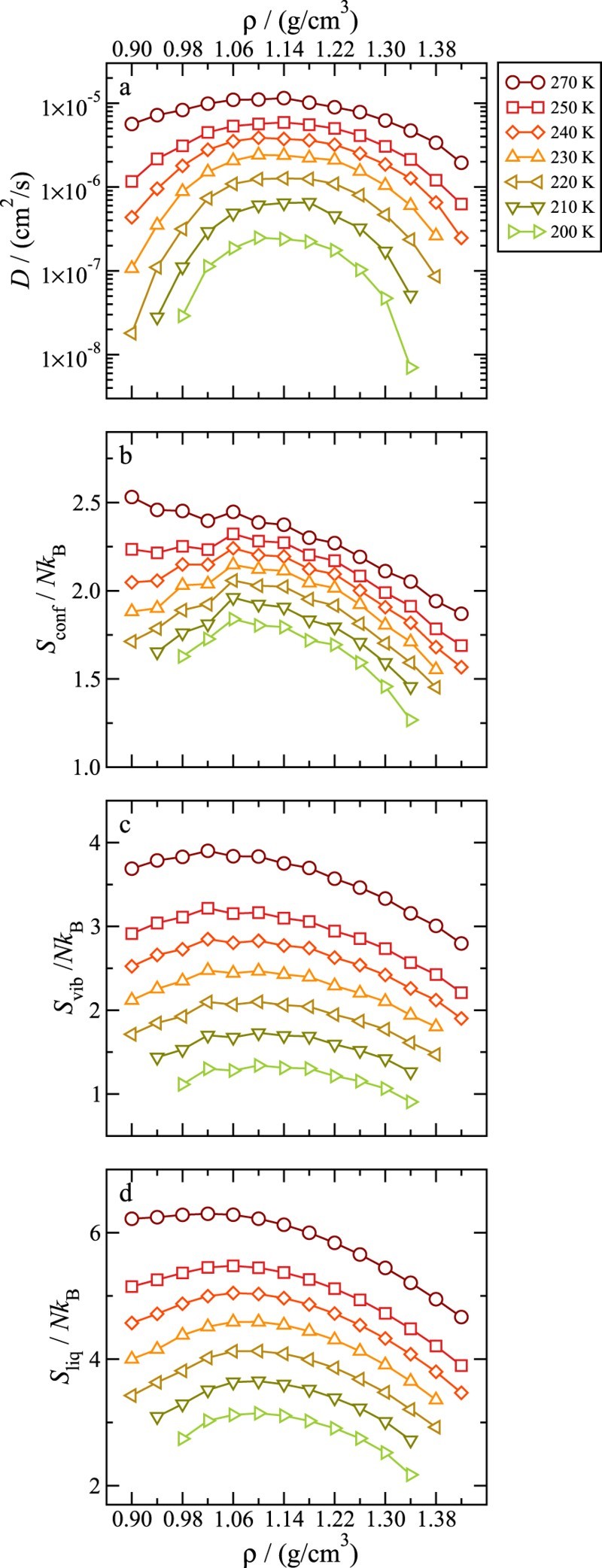
Density dependence of the diffusion constant (a), the configurational entropy (b), the vibrational entropy (c) and the liquid entropy (d). The data are shown for all studied densities and temperatures.

Figure 1 shows the self-diffusion constant as well as the calculated entropies for all studied *T* and *ρ*. All quantities show maxima in their respective *ρ* dependence, consistent with the unconventional dynamic and thermodynamic behaviour of water. As already pointed out [20] the maximum in $S_{\mathrm{conf}}$ can be explained as a balance of two mechanisms. At low *ρ* the structure of the liquid resembles the one of a tetrahedral network which is expected to be characterised by a small number of potential energy minima and therefore a small $S_{\mathrm{conf}}$. On increasing *ρ*, the network is progressively distorted increasing the number of potential energy minima and thereby $S_{\mathrm{conf}}$. At even higher *ρ* the Lennard-Jones repulsion becomes dominant, decreasing the number of minima and $S_{\mathrm{conf}}$. This non monotonic behaviour of $S_{\mathrm{conf}}$ is already evident at high *T*. Indeed, the estimated total number of PEL basins does already show such non monotonic behaviour [28]. Also the maxima in $S_{\mathrm{liq}}$ and *D* are consistent with the previous results on the SPC/E model of water [20]. The observed maximum in $S_{\mathrm{vib}}$ in Figure 1 however contrasts the result for SPC/E, where no maximum was reported.

**Figure 2.**
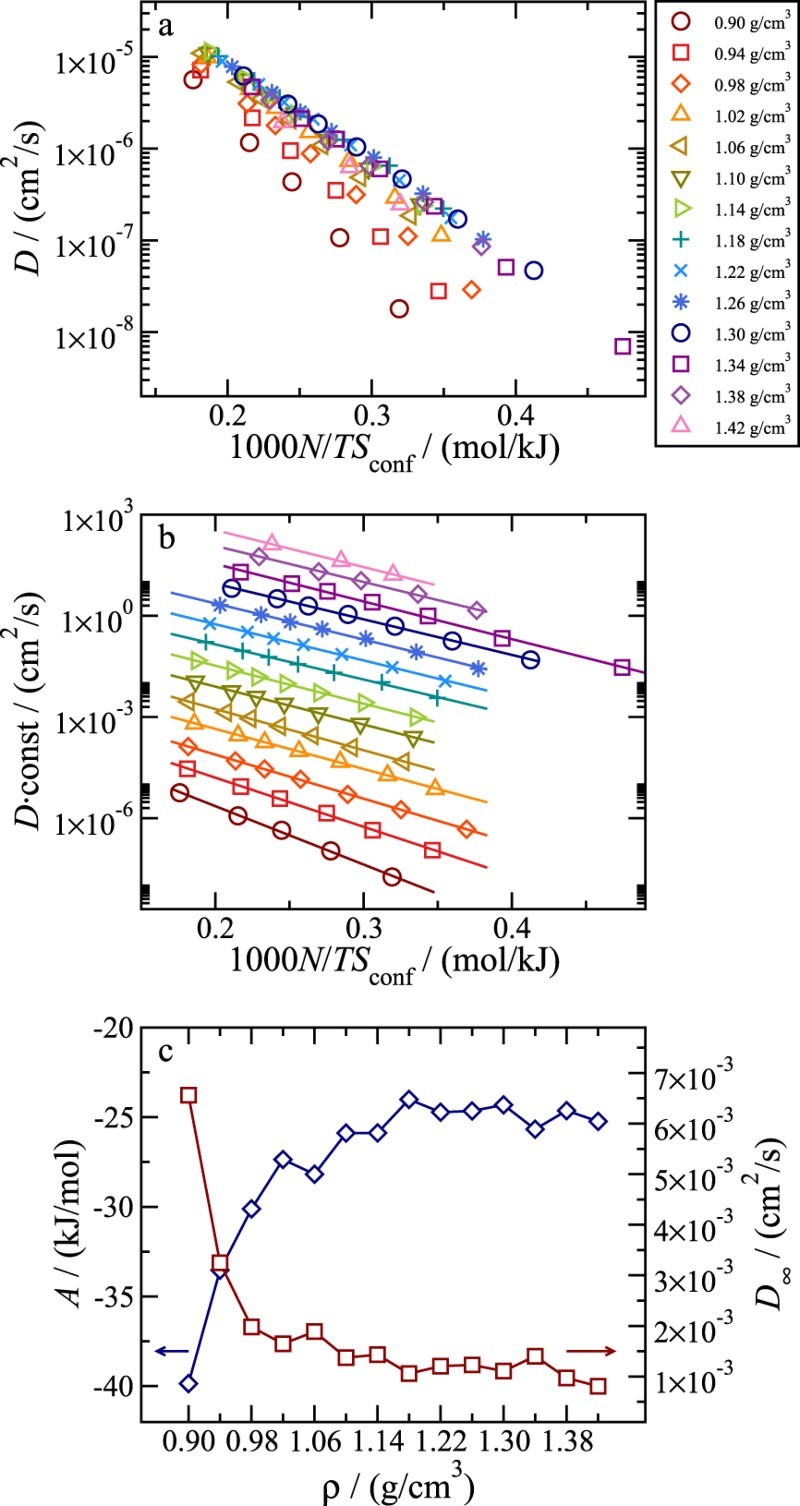
Semi-log plot of the diffusion constant D vs. 1/$TS_{\mathrm{conf}}$ for all studied state points. Part (a) shows the data as obtained and part (b) shows vertically shifted data. Here the solid lines represent best fits with the Adam–Gibbs relation (Equation (2)). Part (c) shows the parameters obtained by fitting the data shown in (a) with Equation (2). The main axis shows the parameter *A* and the alternative axis the pre-exponential constant $D_{\infty }$.

From the *T* dependence of both $S_{\mathrm{conf}}$ and *D* it is possible to check the validity of the Adam–Gibbs relation (Equation  (2)). Figure 2(a) shows ln⁡(D) vs. $1/TS_{\mathrm{conf}}$. The same data are also reported in Figure 2(b) arbitrarily shifted in the *y* direction to highlight the linear dependence, consistent with the theoretical prediction of the Adam–Gibbs relation. The density dependence of the parameters *A* and $D_{\infty }$ are shown in Figure 2(c). Both *A* and $D_{\infty }$ appear to approach a density independent value at large *ρ*. Significant deviations are only visible at low *ρ*, in the region where a well connected network of hydrogen bonded molecules develops. We note on passing that if the thermal velocity $\sqrt{k_{B}T/M}$ is explicitly accounted for by normalising *D*, the quality of the AG fit remains identical.

For completeness, we compare the TIP4P/2005 data also with the excess entropy scaling [31,41–47] proposition which relates *D* with the excess entropy $S_{\mathrm{exc}}$. The rationale behind this hypothesis is that dynamics is controlled by the total number of accessible microstates in phase space. The validity of such a scaling can be tested if an adimensional diffusion constant $D^{*}$ is plotted against $S_{\mathrm{exc}}$. $D^{*}$ is commonly defined as [42]

(17) $$D^{*}=\frac{D\rho^{1/3}}{\sqrt{k_{B}T/M}},$$

to scale out the the trivial thermal velocity contribution $\sqrt{k_{B}T/M}$. Here *ρ* is the molecule number density and *M* the molecular mass.

**Figure 3.**
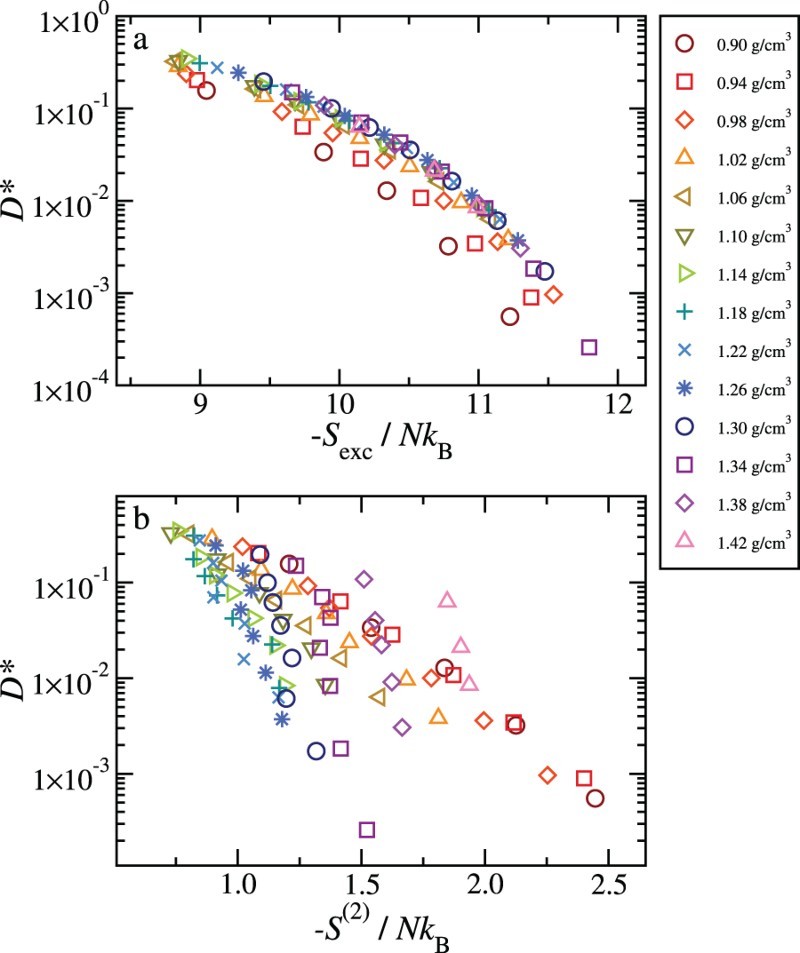
Plots of the reduced diffusion constant versus $S_{\mathrm{exc}}$ (a) and $S^{\left(2\right)}$ (b).

We summarise the corresponding results in Figure 3(a). The data clearly show that there is no data collapse for densities lower than 1.1 g/cm^3^. A reasonable collapse is observed for 1.1<ρ<1.3 only, i.e., in the region where the hydrogen bond network is significantly distorted. Finally we note that if one approximates the excess entropy with the translational contribution $S^{\left(2\right)}$ (see Figure3(b)) an even worse scaling is observed, in accordance with the results of Chopra *et al*. [31] for SPC/E.

## 4. Conclusions

We have recently reported [28] an exhaustive investigation of the potential energy landscape of TIP4P/2005, one of the most accurate classic water models. This model is able to reproduce the complex pattern of thermodynamic anomalies which characterise liquid and supercooled water [48]. In that study an accurate evaluation of the number of PEL basins sampled at each *T* and *ρ* has been carried out, providing access to the configurational entropy. Here we combine the evaluated diffusion coefficient with the associated configurational and excess entropy to test the validity of the Adam–Gibbs relation and of the excess entropy scaling. Despite the complexity of the system (which is characterised by non-monotonic dependence of the diffusion coefficient on the density), ln⁡D is linear in $1/TS_{\mathrm{conf}}$ for all densities. The present data, and the previously available data for the SPC/E model [20], provide strong support to the hypothesis that the number of PEL basins is a key quantity in controlling the dynamics in supercooled water.

## References

[1] Gibaud T., Mahmoudi N., Oberdisse J., Lindner P., Pedersen J.S., Oliveira C.L., Stradner A. and Schurtenberger P., Faraday Discuss. 158, 267 (2012). doi: 10.1039/c2fd20048a 23234170

[2] Asherie N., Methods 34, 266 (2004). doi: 10.1016/j.ymeth.2004.03.028 15325646

[3] Debenedetti P.G., *Metastable Liquids: Concepts and Principles* (Princeton University Press, Princeton, 1996)

[4] Burton E. and Oliver W.F., Nature 135, 505 (1935). doi: 10.1038/135505b0

[5] Brüggeller P. and Mayer E., Nature 288, 569 (1980). doi: 10.1038/288569a0

[6] Mishima O., Calvert L.D. and Whalley E., Nature 310, 393 (1984). doi: 10.1038/310393a0

[7] Handle P.H. and Loerting T., Phys. Chem. Chem. Phys. 17, 5403 (2015). doi: 10.1039/C4CP05587J 25613472

[8] Angell C.A., Science 267, 1924 (1995). doi: 10.1126/science.267.5206.1924 17770101

[9] Adam G. and Gibbs J.H., J. Chem. Phys. 43, 139 (1965). doi: 10.1063/1.1696442

[10] Giovambattista N., Buldyrev S.V., Starr F.W. and Stanley H.E., Phys. Rev. Lett. 90, 085506 (2003). doi: 10.1103/PhysRevLett.90.085506 12633440

[11] Dyre J.C., Hechsher T. and Niss K., J. Non-Cryst. Solids 355, 624 (2009). doi: 10.1016/j.jnoncrysol.2009.01.039

[12] Stillinger F.H., *Energy Landscapes, Inherent Structures, and Condensed-matter Phenomena* (Princeton University Press, Princeton, 2015)

[13] Sciortino F., Kob W. and Tartaglia P., Phys. Rev. Lett. 83, 3214 (1999). doi: 10.1103/PhysRevLett.83.3214

[14] Sciortino F., Kob W. and Tartaglia P., J. Phys. Condens. Matter. 12, 6525 (2000). doi: 10.1088/0953-8984/12/29/324

[15] Sastry S., J. Phys. Condens. Matter. 12, 6515 (2000). doi: 10.1088/0953-8984/12/29/323

[16] Sciortino F., J. Stat. Mech. 2005, P05015 (2005). doi: 10.1088/1742-5468/2005/05/P05015

[17] Greet R. and Turnbull D., J. Chem. Phys. 47, 2185 (1967). doi: 10.1063/1.1712251

[18] Richert R. and Angell C., J. Chem. Phys. 108, 9016 (1998). doi: 10.1063/1.476348

[19] Roland C., Capaccioli S., Lucchesi M. and Casalini R., J. Chem. Phys. 120, 10640 (2004). doi: 10.1063/1.1739394 15268090

[20] Scala A., Starr F.W., La Nave E., Sciortino F. and Stanley H.E., Nature 406, 166 (2000). doi: 10.1038/35018034 10910351

[21] Sastry S., Nature 409, 164 (2001). doi: 10.1038/35051524 11196634

[22] Speedy R.J., J. Chem. Phys. 110, 4559 (1999). doi: 10.1063/1.478337

[23] Angelani L. and Foffi G., J. Phys. Condens. Matter 19, 256207 (2007). doi: 10.1088/0953-8984/19/25/256207

[24] Mossa S., La Nave E., Stanley H., Donati C., Sciortino F. and Tartaglia P., Phys. Rev. E 65, 041205 (2002). doi: 10.1103/PhysRevE.65.041205 12005814

[25] Saika-Voivod I., Sciortino F. and Poole P.H., Phys. Rev. E 69, 041503 (2004). doi: 10.1103/PhysRevE.69.041503 15169021

[26] Starr F.W., Douglas J.F. and Sastry S., J. Chem. Phys. 138, 12A541 (2013). doi: 10.1063/1.4790138 PMC3598772 23556792

[27] Parmar A.D. and Sastry S., J. Phys. Chem. B 119, 11243 (2015). doi: 10.1021/acs.jpcb.5b03122 26134744

[28] Handle P.H. and Sciortino F., J. Chem. Phys. 148, 134505 (2018). 29626874 10.1063/1.5023894

[29] Abascal J.L. and Vega C., J. Chem. Phys. 123, 234505 (2005). doi: 10.1063/1.2121687 16392929

[30] Vega C. and Abascal J.L., Phys. Chem. Chem. Phys. 13, 19663 (2011). doi: 10.1039/c1cp22168j 21927736

[31] Chopra R., Truskett T.M. and Errington J.R., J. Phys. Chem. B 114, 10558 (2010). doi: 10.1021/jp1049155 20701386

[32] Van Der Spoel D., Lindahl E., Hess B., Groenhof G., Mark A.E. and Berendsen H.J., J. Comput. Chem. 26, 1701 (2005). doi: 10.1002/jcc.20291 16211538

[33] Nosé S., Mol. Phys. 52, 255 (1984). doi: 10.1080/00268978400101201

[34] Hoover W.G., Phys. Rev. A 31, 1695 (1985). doi: 10.1103/PhysRevA.31.1695 9895674

[35] Essmann U., Perera L., Berkowitz M.L., Darden T., Lee H. and Pedersen L.G., J. Chem. Phys. 103, 8577 (1995). doi: 10.1063/1.470117

[36] Hess B., J. Chem. Theory Comput. 4, 116 (2008). doi: 10.1021/ct700200b 26619985

[37] Frenkel D., Smit B., *Understanding Molecular Simulation: From Algorithms to Applications* (Elsevier, San Diego, 2001)

[38] Frenkel D. and Ladd A.J., J. Chem. Phys 81, 3188 (1984). doi: 10.1063/1.448024

[39] Vega C., Sanz E., Abascal J. and Noya E., J. Phys. 20, 153101 (2008).

[40] Mayer J.E., Mayer M.G., *Statistical Mechanics* (John Wiley & Sons, New York, 1963)

[41] Rosenfeld Y., Phys. Rev. A 15, 2545 (1977). doi: 10.1103/PhysRevA.15.2545

[42] Rosenfeld Y., J. Phys. Condens. Matter 11, 5415 (1999). doi: 10.1088/0953-8984/11/28/303

[43] Dzugutov M., Nature 381, 137 (1996). doi: 10.1038/381137a0

[44] Dzugutov M., Aurell E. and Vulpiani A., Phys. Rev. Lett. 81, 1762 (1998). doi: 10.1103/PhysRevLett.81.1762

[45] Hoyt J., Asta M. and Sadigh B., Phys. Rev. Lett. 85, 594 (2000). doi: 10.1103/PhysRevLett.85.594 10991348

[46] Sharma R., Chakraborty S.N. and Chakravarty C., J. Chem. Phys. 125, 204501 (2006). 17144709 10.1063/1.2390710

[47] Chakraborty S.N. and Chakravarty C., J. Chem. Phys. 124, 014507 (2006). 10.1063/1.2140282 16409041

[48] Handle P.H., Loerting T. and Sciortino F., Proc. Natl. Acad. Sci. U.S.A. 201700103 (2017). 10.1073/pnas.1700103114 PMC5754753 29133419

